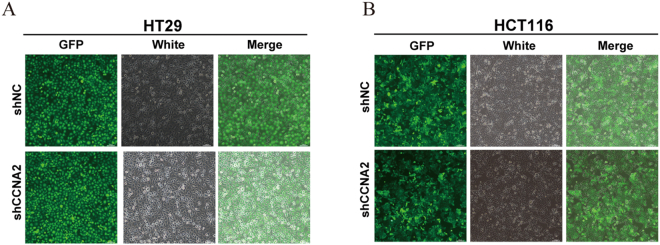# Exploring the regulatory mechanism of *CCNA2* in colorectal cancer: Insights from multiomics and experimental analysis

**DOI:** 10.1016/j.jbc.2025.110216

**Published:** 2025-05-08

**Authors:** Xinyi Lei, Lanying Qiu, Qiang Chen, Lan Liao, Pengfei Yu, Wenjie Wu, Zhengyang Zhu, Chunying Li, Gang Lin, Zirui Zhuang, Yuxin Meng, Yan Wang, Cunchuan Wang, Yian Du

**Affiliations:** 1 Department of Gastric Surgery, Zhejiang Cancer Hospital, Hangzhou Institute of Medicine (HIM), Chinese Academy of Sciences, Hangzhou, Zhejiang, China; 2 Department of Gastrointestinal Surgery, The First Affiliated Hospital of Jinan University, Guangzhou, Guangdong, China; 3 Department of Chest Radiotherapy, Zhejiang Cancer Hospital, Hangzhou Institute of Medicine (HIM), Chinese Academy of Sciences, Hangzhou, Zhejiang, China; 4 Department of Oncology, Cancer Diagnosis and Therapy Research Center, The First Affiliated Hospital of Jinan University, Guangzhou, Guangdong, China; 5 Department of Pathology, The First Affiliated Hospital, University of South China, Hengyang, Hunan, China; 6 Department of Radiation Physics, Zhejiang Cancer Hospital, Hangzhou Institute of Medicine (HIM), Chinese Academy of Sciences, Hangzhou, Zhejiang, China; 7 Department of Integrated Traditional Chinese and Western Medicine, Zhejiang Cancer Hospital, Hangzhou Institute of Medicine (HIM), Chinese Academy of Sciences, Hangzhou, Zhejiang, China; 8 Zhejiang Cancer Hospital, Hangzhou Institute of Medicine (HIM), Chinese Academy of Sciences, Hangzhou, Zhejiang, China; 9 School of Molecular Medicine, Hangzhou Institute for Advanced Study, University of Chinese Academy of Sciences (UCAS), Hangzhou, Zhejiang, China; 10 Shanghai Institute of Materia Medica, Chinese Academy of Sciences, Shanghai, China; 11 College of Pharmaceutical Science, Zhejiang University of Technology, Hangzhou, Zhejiang, China; 12 Collaborative Innovation Center of Yangtza River Delta Region Green Pharmaceuticals, Zhejiang University of Technology, Hangzhou, Zhejiang, China

**Keywords:** *CCNA2*, colorectal cancer, multiomics, experimental analysis, miR-548x-3p

## Abstract

Colorectal cancer (CRC) is the third-most common cancer and the second leading of cancer-related deaths worldwide. The underlying regulatory mechanism of cyclin A2 (*CCNA2*) in CRC was explored through multiomics and experimental analyses, thus facilitating diagnosis, therapy, and prognosis. GSE9348 and GSE110223 were extracted from Gene Expression Omnibus. Differentially expressed genes (DEGs) were identified *via* GEO2R. *CCNA2*, a core gene for CRC, was screened out from the protein–protein interaction network constructed by differentially expressed genes. Its diagnostic, prognostic, and therapeutic value was evaluated in Gene Expression Omnibus, The Cancer Genome Atlas, Human Protein Atlas, and Drug-Gene interaction database via transcriptomics, proteomics, and pharmacogenomics. The correlation between *CCNA2* and immune infiltration was determined in Tumor Immune Estimation Resource by immunomics. Transcription factor–mRNA and miRNA–mRNA networks for *CCNA2* were constructed in miRnet and miRDB *via* transcriptomics. The role and regulatory mechanism of *CCNA2* in CRC were investigated both *in vitro* and *in vivo*. *CCNA2* showed excellent diagnostic, therapeutic, and prognostic value in CRC. *CCNA2* was closely associated with tumor-infiltrating immunocytes, transcription factors, and miRNAs. The knockdown of *CCNA2* inhibited the proliferation, migration, invasion, epithelial–mesenchymal transition (EMT), while inducing apoptosis of CRC cells. *CCNA2* acted as a target of miR-548x-3p in regulating the biological behavior of CRC cells *via* the EMT-signaling pathway. *CCNA2* is a potential biomarker for the diagnosis, treatment, and prognosis of CRC. The miR-548x-3p–*CCNA2* axis plays a pivotal role in regulating the tumorigenesis of CRC through the EMT-signaling pathway.

Globally, colorectal cancer (CRC) is the third-most common type of malignancy and the second-leading cause of mortality because of cancer (1). More than 1,800,000 new CRC cases and 915,880 deaths were estimated to occur in 2020, representing almost 9% of all cancer cases and deaths (2). The incidence rates of CRC have steadily increased in Eastern Europe, South Eastern, South Central Asia, and South America (3, 4). In recent years, the incidence of CRC has declined in some high-incidence countries owing to lifestyle changes and uptake of early screening (5), suggesting that the prognosis of CRC can be improved by early screening. However, the screening and validation of core biomarkers in CRC remains a great challenge.

Both genetic and epigenetic alterations are involved in modulating the initiation and development of CRC (6). Cyclin A2 (*CCNA2*) is a core cell cycle regulator in the cyclin family. In mice, *CCNA2* is essential for early embryogenesis, and disrupting its gene can result in early embryonic lethality (7). During early mitosis, *CCNA2* is involved in mitotic spindle anchoring and correcting the aberrant kinetochore–microtubule attachments (8, 9). In recent years, some studies confirmed that the expression of *CCNA2* increased in breast cancer and ovarian cancer (10, 11). Guo *et al*. (12) conducted a meta-analysis of transcriptome datasets containing primary CRC samples and normal samples, which revealed increased *CCNA2* transcript levels in primary CRC samples, when compared with those in normal samples. Yang *et al*. (13) reported that farnesoid X receptor induced miR-22 in the suppression of *CCNA2*, which inhibited the proliferation of CRC cells. Thus, the role and regulatory mechanism of *CCNA2* in CRC warrant further studies.

The development of biochemical techniques, particularly next-generation sequencing, has allowed the systematic analysis of the genomic characteristics of carcinomas. Large-scaled, multiomics analyses of carcinomas have given novel perspectives on the dysregulation of cancer genes (14). Large-scale, public, and multiomics databases were applied for multiomic analyses of the occurrence and development of carcinomas in terms of genomics, epigenomics, transcriptomics, and proteomics. Gene Expression Omnibus (GEO) was constructed by the National Center for Biotechnology Information, which provides flexible and open heterogeneous datasets from high-throughput gene expression and genomic hybridization experiments (15). The Cancer Genome Atlas (TCGA) has compiled large high-dimensional datasets across different types of cancers and molecular platforms. TCGA has led to great progress in molecular subtype identification and also improved the diagnosis and prognosis of cancers (16).

miRNA-conserved small noncoding RNAs (18–25 nt) are generated from endogenous hairpin–shaped precursors, which can directly bind to specific sites presented in the 3′-UTRs of the target mRNA, which led to either mRNA decay or translational blockade by the formation of RNA-induced silencing complex (17). A previous study showed increased expressions of miRNA-135a and miRNA-135b in CRC patients who showed decreased expression of APC (18). The downregulation of miR-200a, miR-220b, miR-220c, miR-141, and miR-429 was predicted to be important events for metastasis in CRC (19). Xu *et al*. (20) studied miRNAs and *CCNA2* in the physiological aging mouse *via* high-throughput analysis and noted the senescence-induction effect of miR-124 targeting *CCNA2*. A decreased miR-124 level could increase the *CCNA2* expression in cells and animal models of Huntingtin, which was involved in the deregulation of the cell cycle in STHdh (Q111)/Hdh (Q111) cells (21).

With the application of omics analysis in tumor-related studies, we elucidated the potential underlying regulatory mechanisms in the genesis and progression of CRC from multiple dimensions. In the present study, two gene expression datasets (*i*.*e*., GSE9348 and GSE110223) were combined, for the first time, for integrative analysis in GEO. Differentially expressed genes (DEGs) were identified in CRC samples and compared with those in normal samples. The Database for Annotation, Visualization, and Integrated Discovery (DAVID) was used for functional enrichment analysis of DEGs derived from high-throughput genomics. The protein–protein interaction (PPI) network for DEGs was built through proteomics, and the core genes were identified. The prognostic value of *CCNA2* was identified in CRC *via* transcriptomics and proteomics, respectively. The diagnostic value of *CCNA2* in distinguishing CRC from normal tissues was evaluated based on high-throughput expression profiling data. The therapeutic value of *CCNA2* was determined through pharmacogenomics. The correlation between *CCNA2* and immune infiltration was revealed *via* immunomics. The transcription factor (TF)–mRNA and miRNA–mRNA interaction network for *CCNA2* were constructed based on transcriptomics. The role and regulatory mechanism of *CCNA2* in CRC were verified both *in vitro* and *in vivo*. Our study revealed that miR-548x-3p had a targeted regulatory relationship with *CCNA2*. However, the targeted regulation relationship between miR-548x-3p and *CCNA2* in CRC remains to be fully elucidated. To the best of our knowledge, the present study is the first time to reveal the regulatory mechanism of miR-548x-3p–*CCNA2* axis in regulating tumorigenesis of CRC through the epithelial–mesenchymal transition (EMT) signaling pathway. The results of the present study were acquired through multiomics and experimental analyses, thus facilitating the early diagnosis and precision therapy for CRC.

## Results

### Identification of core genes in CRC

**Figure 1:**
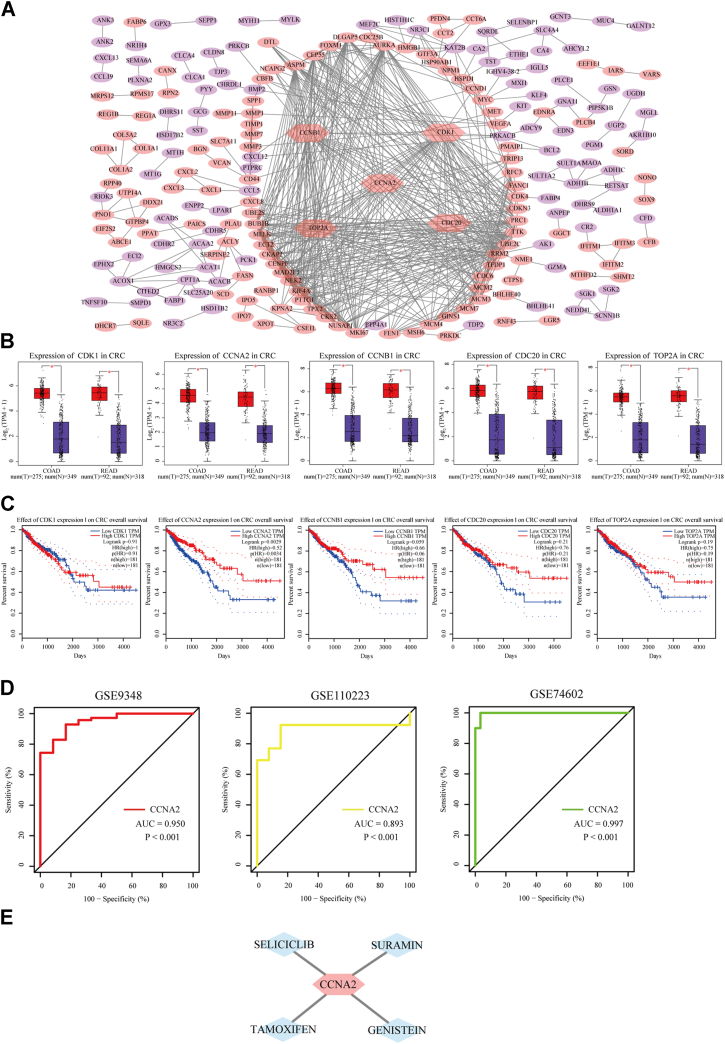
**Identification of core genes in CRC**. *A*, the protein–protein interaction (PPI) network for DEGs. *Red nodes* represented upregulated DEGs, *purple nodes* represented downregulated DEGs, and *hexagonal nodes* represented core genes. *B*, the mRNA expressions of core genes in CRC were verified *via* GEPIA based on TCGA and GTEx (Genotype-Tissue Expression). *C*, the overall survival analysis of core genes was performed through GEPIA based on TCGA. Based on the transcripts per million (TPM) of core genes, the clinical data of CRC patients were divided into two groups: the high and low groups’ TPM were higher and lower than the median value, respectively. *D*, ROC analysis of prognostic core gene expressions for CRC in internal datasets (GSE9348 and GSE110223) and external dataset (GSE74602). *E*, the drug–gene interaction network for prognostic core gene. *Hexagonal node* represented the prognostic core gene; *diamond nodes* represented drugs. COAD, colon adenocarcinoma; CRC, colorectal cancer; DEG, differently expressed gene; READ, rectum adenocarcinoma; ROC, receiver operating characteristic; TCGA, The Cancer Genome Atlas.

**Figure S1:**
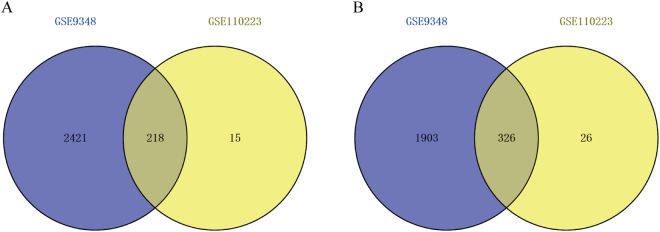


**Figure S2:**
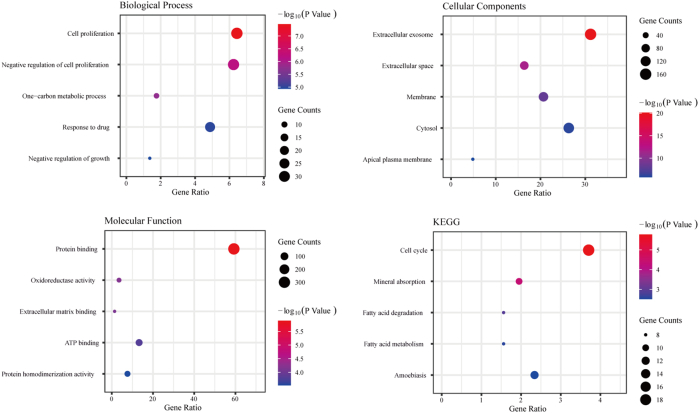


**Figure S3:**
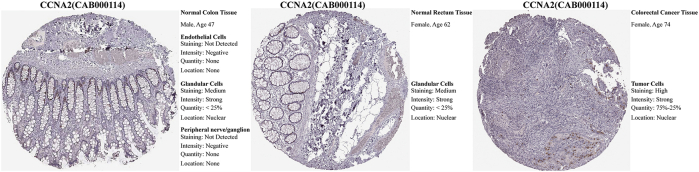


A total of 4868 and 585 DEGs were filtered from GSE9348 and GSE110223, respectively. The expression trends of 544 DEGs shared between the two gene datasets are shown in Figure S1. They included 218 upregulated and 326 downregulated DEGs (Table S1). The top five Gene Ontology terms and Kyoto Encyclopedia of Genes and Genomes pathways enriched by DEGs are shown in Figure S2 (ranked by −log_10_ [*p* value]). From 544 DEGs, 231 DEGs were selected to construct the PPI network. The PPI network covered 127 upregulated DEGs, 104 downregulated DEGs, and 633 interactions (Fig. 1*A*). The DEGs ranked in the top five based on the degree (*CDK1* [cyclin-dependent kinase 1], *CCNA2*, *CCNB1*, *CDC20*, and *TOP2A*) were considered core genes for CRC (Table S1 is bold). The characteristics of core genes are shown in Table S2. The mRNA expressions of *CDK1*, *CCNA2*, *CCNB1*, *CDC20*, and *TOP2A* were higher in CRC samples than in normal samples (Fig. 1*B*). The results of the survival analysis showed that the higher mRNA expression of *CCNA2* was associated with the higher survival rates in CRC. This indicated that *CCNA2* had a prognostic value in CRC, and *CCNA2* is the prognostic core gene in CRC (Fig. 1*C*). *CCNA2* as a positive prognostic indicator for CRC might be related to the source of patients, the aggressiveness of therapy, the initiation of the anticancer process, and so on. In Human Protein Atlas, the protein expression of CCNA2 was higher in CRC tissues than in normal colorectal tissues (Fig. S3). *CCNA2* showed excellent diagnostic value in distinguishing between CRC tissues and normal tissues in internal and external datasets (Fig. 1*D*). In the drug–gene interaction network, seliciclib, suramin, tamoxifen, and genistein interacted with *CCNA2* (Fig. 1*E*).

### Bioinformatics analysis of prognostic core gene in CRC

**Figure 2:**
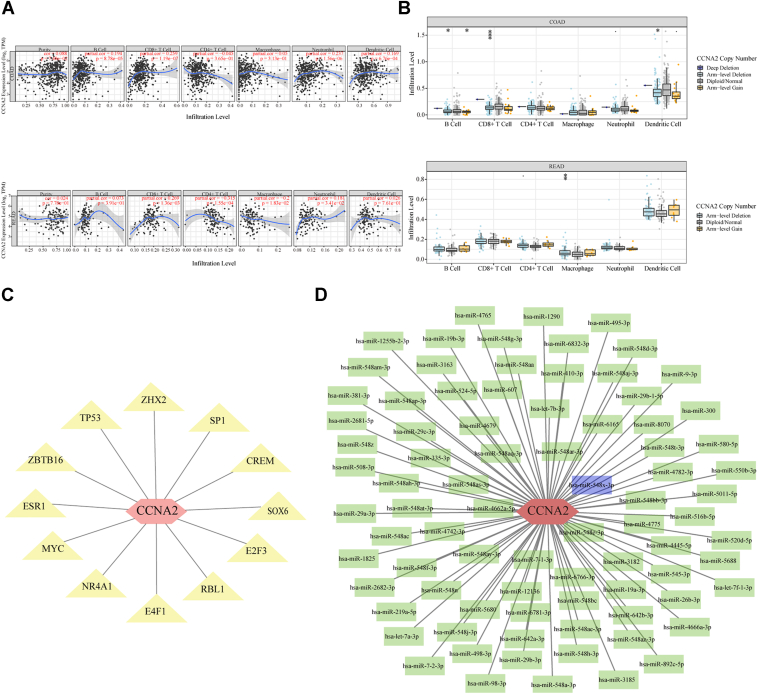
**Bioinformatics analysis of prognostic core gene in CRC**. *A*, correlation between expression of prognostic core gene and infiltration levels of immunocytes in CRC. *B*, correlation between somatic copy number alterations (SCNAs) of prognostic core gene and infiltration levels of immunocytes in CRC. *C*, the transcription factor (TF)–mRNA interaction network for prognostic core gene. *Hexagonal node* represented prognostic core gene; *triangle nodes* represented TFs. *D*, the miRNA–mRNA interaction network for prognostic core gene. *Hexagonal node* represented prognostic core gene; *rectangle nodes* represented miRNAs. COAD, colon adenocarcinoma; CRC, colorectal cancer; READ, rectum adenocarcinoma.

Immunotherapy has significantly changed clinical management of CRC (22). Efficient biomarkers were needed as predictors for responsiveness of immunotherapy. *CCNA2* has been proved as a potential target for tumor immunotherapy and high-avidity *CCNA2*-specific cytotoxic T lymphocyte could be generated using CD40-B cells as antigen-presenting cells (23). In colon adenocarcinoma, mRNA expression of *CCNA2* correlated with the infiltration levels of B cell (cor = 0.194, *p* = 8.78e-5), CD8+ T cell (cor = 0.259, *p* = 1.19e-7), neutrophil (cor = 0.237, *p* = 1.56e-6), and dendritic cell (cor = 0.169, *p* = 6.76e-4); in rectum adenocarcinoma, mRNA expression of *CCNA2* correlated with the infiltration levels of CD8+ T cell (cor = 0.269, *p* = 1.36e-3), CD4+ T cell (cor = −0.315, *p* = 1.55e-4), macrophage (cor = −0.200, *p* = 1.83e-2), and neutrophil (cor = 0.181, *p* = 3.41e-2) (Fig. 2*A*). In colon adenocarcinoma, the somatic copy number alterations (SCNAs) of *CCNA2* correlated with the infiltration levels of B cells, CD8+ T cells, and dendritic cells; in rectum adenocarcinoma, somatic copy number alteration (SCNA) of *CCNA2* correlated with the infiltration levels of macrophages (Fig. 2*B*). The association between *CCNA2* and gene markers on different subsets of immune cells in CRC is shown in Table 1. The TF–mRNA interaction network for *CCNA2* contained 12 TFs and *CCNA2* (Fig. 2*C*). The miRNA–mRNA interaction network for *CCNA2* contained 83 miRNAs and *CCNA2* (Fig. 2*D*).

**Table 1:** Correlation analysis between *CCNA2* and gene markers of immunocytes in COAD and READ

Description	Gene marker	CCNA2			
		COAD		READ	
		Cor	*p*	Cor	*p*
CD8+ T cell	CD8A	0.167	c	−0.001	0.988
	CD8B	0.070	0.158	0.019	0.821
T cell (general)	CD2	0.157	b	0.057	0.506
	CD3D	0.063	0.205	0.042	0.624
	CD3E	0.039	0.438	−0.061	0.478
Th1	CD4	0.059	0.234	−0.146	0.086
	IFNG	0.252	c	0.173	a
	STAT1	0.269	c	0.276	b
	STAT4	0.185	c	0.093	0.274
	TBX21	0.145	b	−0.010	0.905
Th2	CCR4	0.070	0.158	−0.042	0.622
	CXCR4	0.084	0.092	−0.231	b
	GATA3	−0.066	0.187	−0.356	c
	STAT6	−0.204	c	−0.057	0.503
Treg	CCR8	0.095	0.055	−0.012	0.884
	FOXP3	0.005	0.928	−0.164	0.054
	STAT5B	−0.069	0.163	−0.037	0.664
	TGFB1	−0.054	0.282	−0.285	c
T-cell exhaustion	PD-1	0.066	0.184	−0.059	0.492
	CTLA4	0.136	b	0.041	0.633
	LAG3	0.158	b	−0.005	0.949
	TIM-3	0.137	b	−0.047	0.586
	GZMB	0.187	c	0.054	0.526
PDL1	CD274	0.221	c	0.178	a
B cell	CD19	−0.049	0.328	−0.056	0.516
	CD79A	−0.072	0.147	−0.053	0.535
Dendritic cell	CD1C	−0.042	0.395	−0.198	a
	CD11C	0.000	0.996	−0.144	0.091
	NRP1	0.166	c	−0.017	0.847
Monocyte	CD86	0.185	c	0.045	0.595
	CD115	−0.029	0.557	−0.235	b
Neutrophils	CCR7	−0.039	0.432	−0.154	0.070
	CD11B	−0.046	0.357	−0.280	c
	CD66B	−0.019	0.698	−0.068	0.429
Natural killer cell	KIR2DL1	0.128	b	−0.043	0.613
	KIR2DS4	0.154	b	0.180	a
	KIR3DL1	0.088	0.078	0.066	0.438
M1 macrophage	COX2	0.225	c	0.234	b
	NOS2	0.174	c	0.236	b
	IRF5	−0.061	0.221	−0.110	0.198
M2 macrophage	CD163	0.118	a	−0.076	0.372
	MS4A4A	0.138	b	−0.068	0.424
	VSIG4	0.060	0.225	−0.153	0.072
Tumor-associated macrophage	CCL2	0.137	b	−0.082	0.338
	CD68	−0.049	0.328	−0.219	b
	IL10	0.147	b	−0.034	0.694

The correlation analysis was adjusted by tumor purity.

a Represented *p* < 0.05.

b Represented *p* < 0.01.

c Represented *p* < 0.001.

### Expression of *CCNA2* in CRC

**Figure 3:**
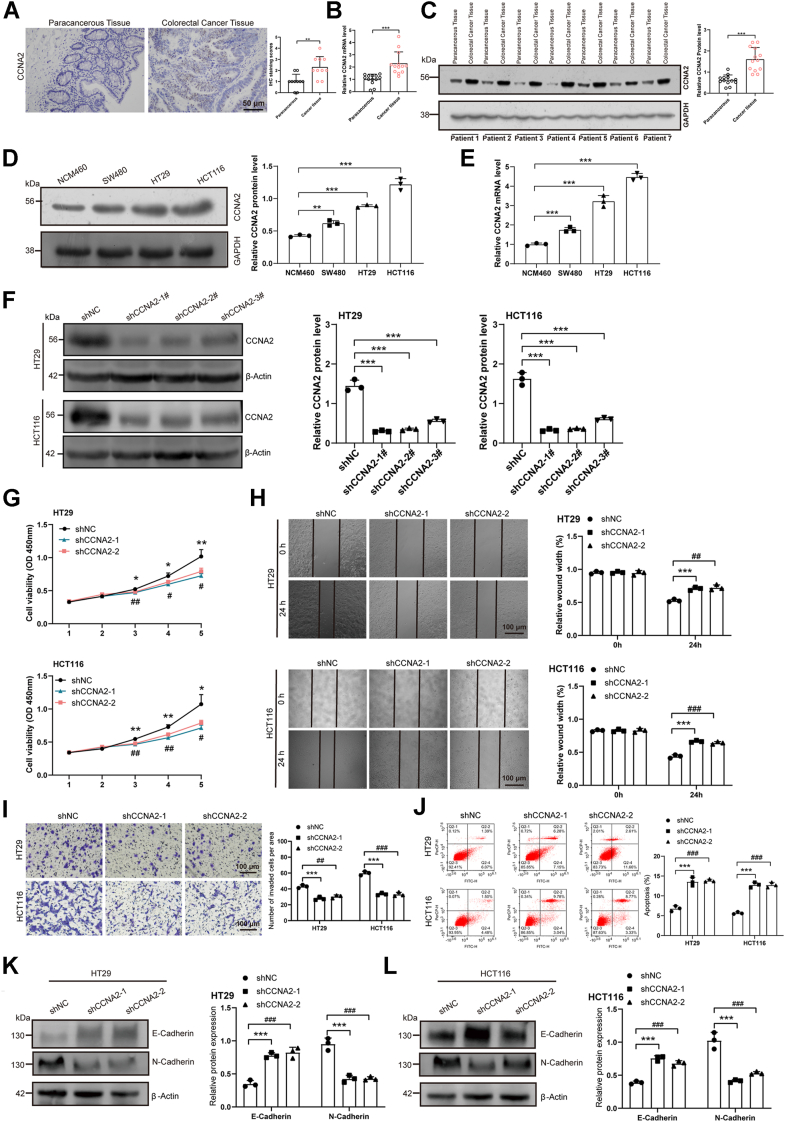
**Expression of *CCNA2* in CRC and its effect on biological behavior of CRC cells**. *A*, the protein expressions of CCNA2 were detected in paracancerous and CRC tissues by immunohistochemistry assay. *B*, the mRNA expressions of *CCNA2* were detected in paracancerous and CRC tissues by quantitative PCR (qPCR) assay. *C*, the protein expressions of CCNA2 were detected in paracancerous and CRC tissues by Western blotting. *D*, the protein expressions of CCNA2 were detected in normal human colonic epithelial and CRC cell lines by Western blotting. *E*, the mRNA expressions of *CCNA2* were detected in normal human colonic epithelial and CRC cell lines by qPCR assay. *F*, the interference efficiency of knockdown *CCNA2* expression in the HCT116 and HT29 stable cell lines was evaluated by Western blotting. *G*, CCK-8 assay showed that knockdown of *CCNA2* expression inhibited the proliferation of HT29 and HCT116 cells. *H*, wound scratch assay demonstrated that hat knockdown of *CCNA2* expression inhibited the migration of HT29 and HCT116 cells. *I*, Transwell assay revealed that knockdown of *CCNA2* expression inhibited the invasion of HT29 and HCT116 cells. *J*, flow cytometry assay indicated that knockdown of *CCNA2* expression induced the apoptosis of HT29 and HCT116 cells. *K*–*L*, the effect of *CCNA2* on the EMT-signaling pathway–associated biomarkers in HT29 and HCT116 cells was analyzed by Western blotting. The data in the bar plots were shown as the mean ± SD. ∗ or # represented *p* < 0.05; ∗∗ or ## represented *p* < 0.01; and ∗∗∗ or ### represented *p* < 0.001. CCK-8, Cell Counting Kit-8; *CCNA2*, cyclin A2; CRC, colorectal cancer; EMT, epithelial–mesenchymal transition.

To determine the role of *CCNA2* in CRC, CRC tissues were subjected to immunohistochemistry, quantitative PCR (qPCR), and Western blotting for the detection of *CCNA2* expression, and paracancerous tissues were used as control. As shown in Figure 3, *A–C*, the results suggested that the expression of *CCNA2* was upregulated in CRC tissues than in paracancerous tissues. Moreover, CRC cell lines (SW480, HT29, and HCT116) were cultured, and a normal colonic mucosal epithelial cell line (NCM460) was used as a control. Cultured cells were subjected to Western blotting and qPCR for the detection of *CCNA2* expression, and the results suggested that *CCNA2* expression was upregulated in CRC cell lines (Fig. 3, *D* and *E*). The aforementioned results revealed that *CCNA2* was upregulated in CRC.

### Effect of *CCNA2* on the biological behavior of CRC *in vitro*

HCT116 and HT29 cell lines with higher expression of *CCNA2* were used to construct stable cell lines with knockdown of *CCNA2*. The Western blotting and fluorescence imaging results showed the knockdown efficiency of *CCNA2* in CRC cells (Fig. 3*F*, Fig. S4, *A* and *B*), which suggested the successful construction of stable cell lines with the knockdown of *CCNA2*. The results of the Cell Counting Kit-8 (CCK-8) assay demonstrated that *CCNA2* knockdown could inhibit the proliferation of HT29 and HCT116 cells (Fig. 3*G*). The wound scratch assay was utilized to determine the migration ability of CRC cells, and the results showed that knockdown of *CCNA2* inhibited the migration of CRC cells (Fig. 3*H*). Furthermore, the results of Transwell experiments showed that the invasive ability of CRC cells was inhibited after *CCNA2* knockdown (Fig. 3*I*). The flow cytometry assay was used to evaluate the apoptosis of CRC cells, and the results suggested that the knockdown of *CCNA2* induced the apoptosis of CRC cells (Fig. 3*J*). These results revealed that *CCNA2* could promote the proliferation, migration, and invasion of CRC cells, as well as inhibit the apoptosis of CRC cells.

During the epithelial–mesenchymal transition, where cells lose their epithelial cell identity and obtain characteristics of mesenchymal cell, represents a salient property of primary tumor formation and metastasis (24). It has been reported that *CCNA2* was involved in the EMT-signaling pathway in different types of diseases (25, 26). However, whether *CCNA2* is involved in the EMT-signaling pathway on CRC is unclear. Therefore, we investigated the regulatory mechanism of *CCNA2* on the EMT-signaling pathway for CRC. Western blotting results demonstrated that the knockdown of *CCNA2* increased the expression of E-cadherin and decreased the expression of N-cadherin in HCT116 and HT29 cell lines (Fig. 3, *K–L*), suggesting that the knockdown of *CCNA2* inhibited the EMT-signaling pathway.

### Expression of miR-548x-3p in CRC

**Figure 4:**
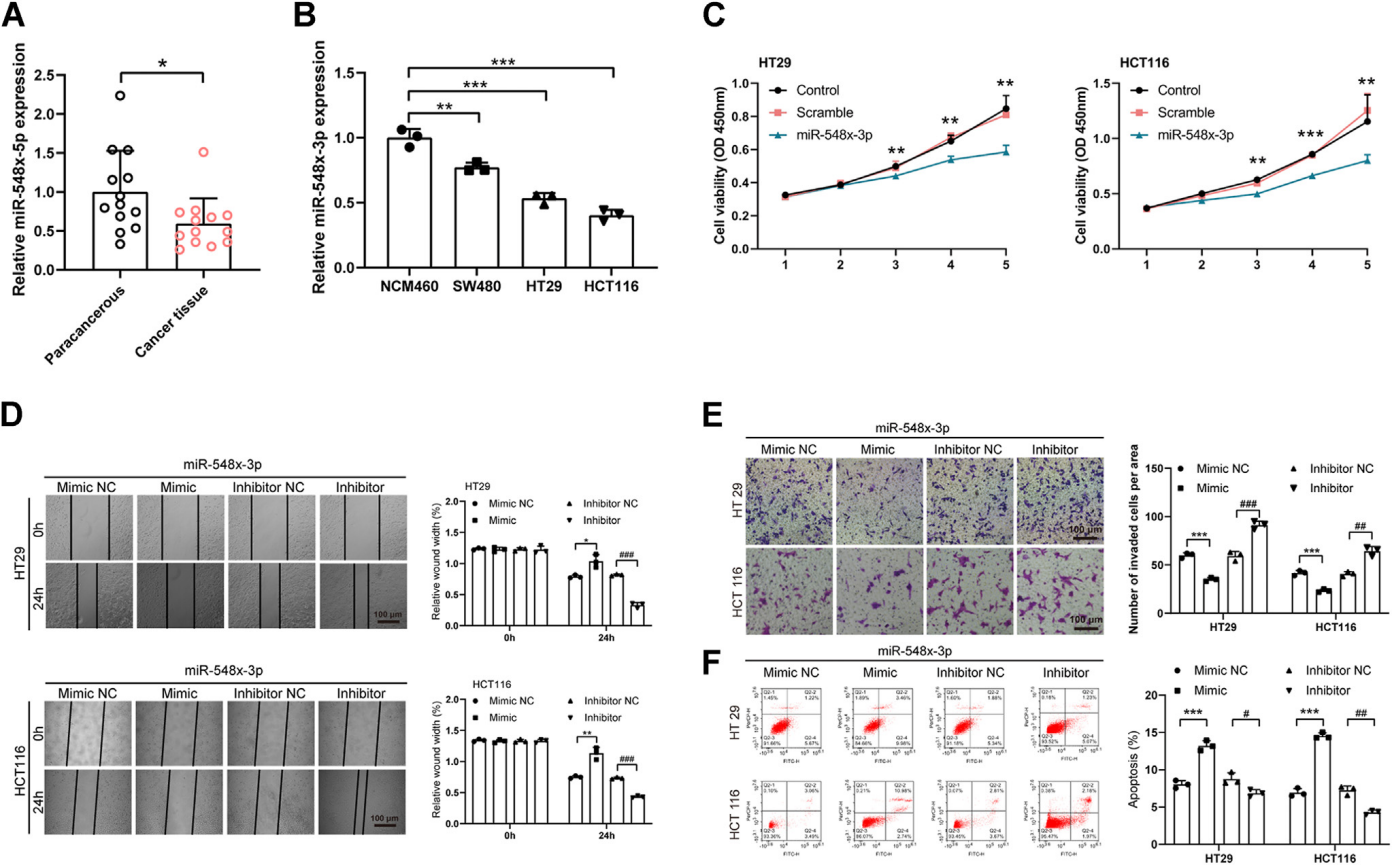
**Expression of miR-548x-3p in CRC and its effect on biological behavior of CRC cells**. *A*, the mRNA expressions of miR-548x-3p were tested in paracancerous and CRC tissues by quantitative PCR (qPCR) assay. *B*, the mRNA expressions of miR-548x-3p were tested in normal human colonic epithelial and CRC cell lines by qPCR assay. *C*, Cell Counting Kit-8 (CCK-8) assay demonstrated that miR-548x-3p overexpression suppressed the proliferation of HT29 and HCT116 cells. *D*, wound scratch assay illustrated that miR-548x-3p overexpression suppressed the migration of HT29 and HCT116 cells. *E*, Transwell assay indicated that miR-548x-3p overexpression suppressed the invasion in HT29 and HCT116 cells. *F*, flow cytometry assay showed that miR-548x-3p overexpression increases apoptosis levels in HT29 and HCT116 cells. The data in the bar plots were shown as the mean ± SD. ∗ or # represented *p* < 0.05; ∗∗ or ## represented *p* < 0.01; and ∗∗∗ or ### represented *p* < 0.001. CRC, colorectal cancer.

Next, the role of miR-548x-3p was determined in CRC. CRC tissues were subjected to qPCR to detect the mRNA expression of miR-548x-3p. Compared with paracancerous tissues, the mRNA expression of miR-548x-3p was downregulated in CRC tissues (Fig. 4*A*). At the cytological level, qPCR results uncovered that the mRNA expression of miR-548x-3p was downregulated in CRC cell lines (HT29, SW480, and HCT116) compared with NCM460 cell line (Fig. 4*B*). The aforementioned results revealed that miR-548x-3p was downregulated in CRC.

### Effect of miR-548x-3p on the biological behavior of CRC

The effect of miR-548x-3p on the proliferation of HT29 and HCT116 cell lines was determined by CCK-8 assay. The results showed that compared with control groups, the proliferation of HT29 and HCT116 cell lines transfected with miR-548x-3p mimic was suppressed (Fig. 4*C*). miR-548x-3p mimic, miR-548x-3p inhibitor, controls fragments as mimic negative control (NC), and inhibitor NC were transfected with HT29 and HCT116 cell lines. The results of wound scratch assays showed that overexpression of miR-548x-3p mimic inhibited the migration of CRC cells. Reversely, overexpression of miR-548x-3p inhibitor promoted the migration of CRC cells (Fig. 4*D*). Moreover, the results of Transwell assays demonstrated that the overexpression of miR-548x-3p mimic suppressed the invasion of CRC cells; correspondingly, the overexpression of miR-548x-3p inhibitor increased the invasion of CRC cells (Fig. 4*E*). The results of flow cytometry assays showed that the overexpression of miR-548x-3p mimic induced the apoptosis of CRC cells (Fig. 4*F*). Based on the aforementioned results, miR-548x-3p could inhibit the proliferation, migration, and invasion of CRC cells, as well as induce the apoptosis of CRC cells.

### Regulation of miR-548x-3p–*CCNA2* axis on the biological behavior of CRC cells

**Figure 5:**
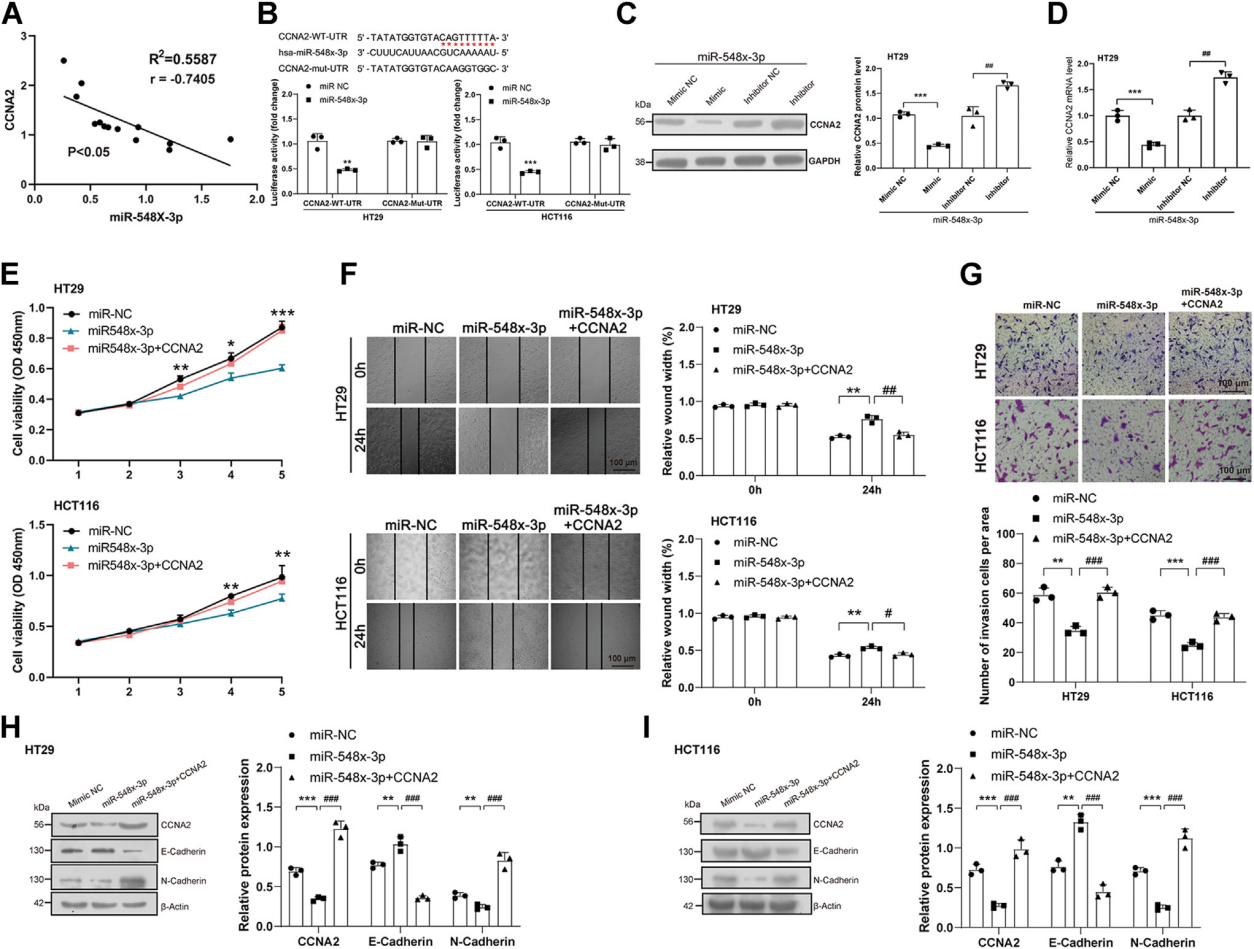
**The miR-548x-3p–*CCNA2* axis regulates the biological behavior of CRC cells**. *A*, correlation analysis of miR-548x-3p and *CCNA2* mRNA expression in CRC. *B*, validation of the targeting relationship between miR-548x-3p and CCNA2 through dual-luciferase reporter assays in CRC cells. *C*, the effect of miR-548x-3p on the targeted regulation of CCNA2 protein expression in CRC cells was analyzed by Western blotting. *D*, the effect of miR-548x-3p on the targeted regulation of *CCNA2* mRNA expression in CRC cells was analyzed by quantitative PCR (qPCR) assay. *E*, CCK-8 assays demonstrated that overexpression of *CCNA2* rescued the inhibitory effect of miR-548x-3p on the proliferation in CRC cells. *F*, wound scratch assay indicated that overexpression of *CCNA2* rescued the inhibitory effect of miR-548x-3p on the migration of CRC cells. *G*, transwell assay showed that overexpression of *CCNA2* rescued the inhibitory effect of miR-548x-3p on the invasion of CRC cells. *H* and *I*, the effect of *CCNA2* on miR-548x-3p regulation of the EMT-signaling pathway–associated biomarkers in CRC cells was analyzed by Western blotting. The data in the bar plots were shown as the mean ± SD. ∗ or # represented *p* < 0.05; ∗∗ or ## represented *p* < 0.01; and ∗∗∗ or ### represented *p* < 0.001. CCK-8, Cell Counting Kit-8; *CCNA2*, cyclin A2; CRC, colorectal cancer; EMT, epithelial–mesenchymal transition.

The targeted regulatory relationship between miR-548x-3p and *CCNA2* was hypothesized by bioinformatics analysis. As shown in Figure 5*A*, the mRNA expression of miR-548x-3p was negatively correlated with that of *CCNA2* in CRC. The dual luciferase reporter assays were conducted to explore the targeted regulatory relationship between miR-548x-3p and *CCNA2*. The results demonstrated that the overexpression of miR-548x-3p inhibited the luciferase signals of the WT *CCNA2* reporter but not that of the MUT *CCNA2* reporter (Fig. 5*B*). Therefore, *CCNA2* is a regulatory target of miR-548x-3p in CRC.

Whether miR-548x-3p regulates CRC development *via CCNA2* was explored in the following assays. The results of Western blotting and qPCR showed that the overexpression of the miR-548x-3p mimic decreased the protein expression and mRNA expression of *CCNA2* in the CRC cells; reversely, the overexpression of the miR-548x-3p inhibitor increased the protein expression and mRNA expression of *CCNA2* in the CRC cells (Fig. 5, *C* and *D*). The results of CCK-8 assays showed that miR-548x-3p overexpression could inhibit CRC cell proliferation; nevertheless, this inhibiting effect could be reversed by *CCNA2* cotransfection (Fig. 5*E*). Similarly, the results of wound scratch assays showed that miR-548x-3p overexpression could suppress CRC cell migration; however, this inhibiting effect could be reversed by *CCNA2* cotransfection (Fig. 5*F*). The same trend could be observed in Transwell assays in the CRC cells (Fig. 5*G*). Based on the aforementioned results, miR-548x-3p regulated the proliferation, migration, and invasion of CRC cells *via* modulating *CCNA2* expression.

The Western blotting results showed that miR-548x-3p overexpression could increase the expression of E-cadherin and decrease the expression of N-cadherin in CRC cells. Nevertheless, this regulatory effect could be reversed by *CCNA2* cotransfection (Fig. 5, *H* and *I*). *CCNA2* could act as a target of miR-548x-3p in regulating the EMT-signaling pathway on CRC.

### Effect of *CCNA2* on the biological behavior of CRC *in vivo*

**Figure 6:**
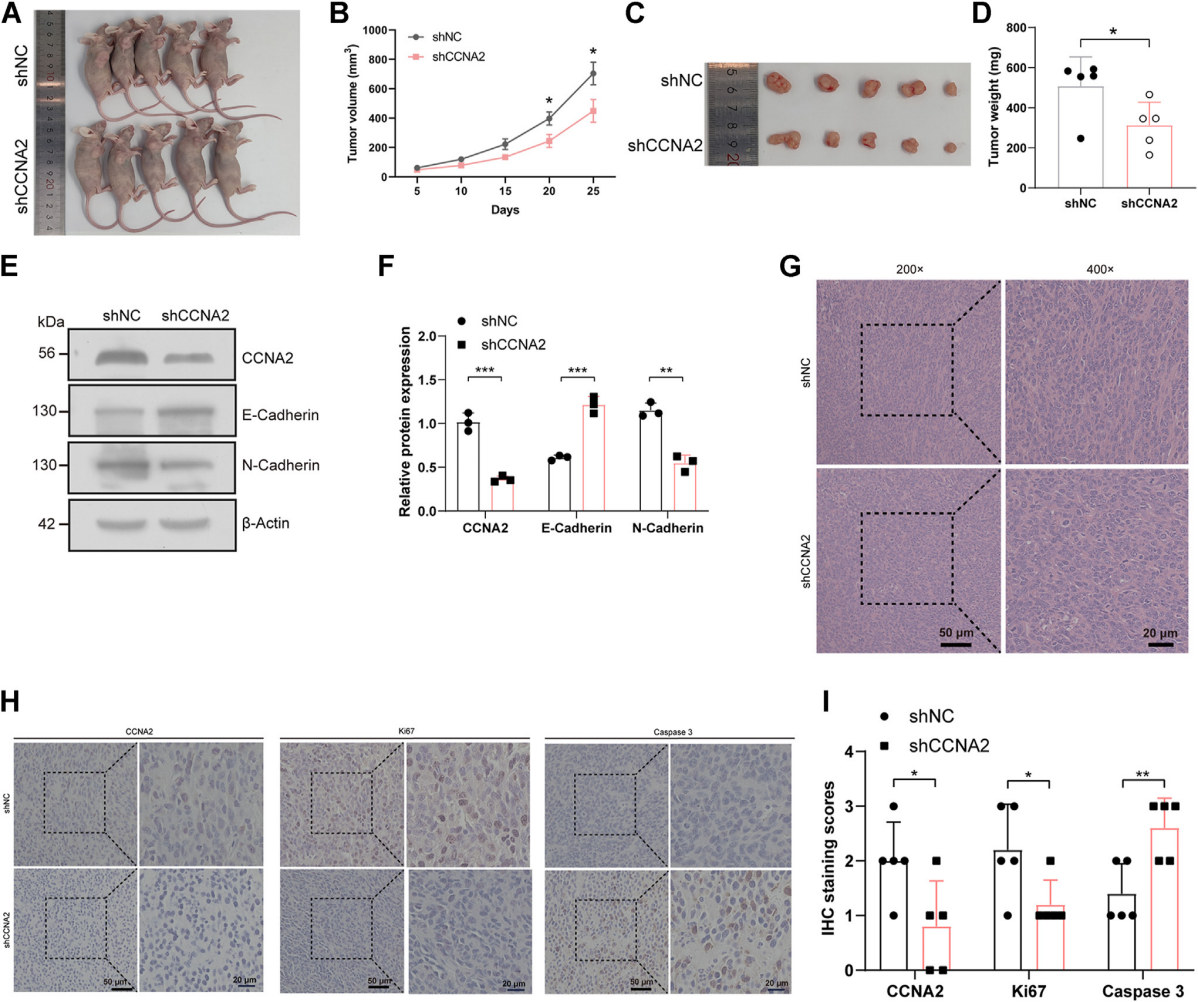
**The effect of *CCNA2* on biological behavior of CRC cells *in vivo***. *A*, the nude mice with subcutaneous tumorigenesis of shNC and sh*CCNA2* CRC cells. *B*, statistical results of subcutaneous tumor xenograft volume in shNC and sh*CCNA2* groups of nude mice. *C*, the appearance of subcutaneous tumor xenografts in shNC and sh*CCNA2* groups of nude mice. *D*, statistical results of subcutaneous tumor xenograft weight in shNC and sh*CCNA2* groups of nude mice. *E* and *F*, the effect of *CCNA2* on the EMT-signaling pathway–associated biomarkers in subcutaneous tumor xenografts was analyzed by Western blotting. *G*, the H&E staining results of subcutaneous tumor xenografts. *H* and *I*, the effect of *CCNA2* on proliferation and apoptosis-associated biomarkers in subcutaneous tumor xenografts was analyzed by immunohistochemistry. The data in the bar plots were shown as the mean ± SD. ∗ represented *p* < 0.05; ∗∗ represented *p* < 0.01; and ∗∗∗ represented *p* < 0.001. *CCNA2*, cyclin A2; CRC, colorectal cancer; EMT, epithelial–mesenchymal transition.

A tumorigenicity assay was performed in nude mice to test the effects of *CCNA2* on the biological behavior of CRC cells *in vivo*. The success rate of subcutaneous tumorigenesis in 10 nude mice was 100% (Fig. 6*A*). The statistical results showed that the volume of tumor xenografts in the *CCNA2* knockdown group was smaller compared with that in the control group (Fig. 6*B*). The appearances of tumor xenografts in the nude mice are shown in Figure 6*C*. The statistical results showed that the weight of tumor xenografts in the *CCNA2* knockdown group was smaller than that in the control group (Fig. 6*D*). Western blotting results revealed that the knockdown of *CCNA2* increased the expression of E-cadherin and decreased the expression of N-cadherin in tumor xenografts (Fig. 6, *E* and *F*). H&E staining results of tumor xenografts are shown in Figure 6*G*. Immunohistochemistry results showed that the knockdown of *CCNA2* decreased the expression of ki67 and increased the expression of caspase 3 in tumor xenografts (Fig. 6, *H* and *I*). Thus, *CCNA2* regulated the development of CRC *via* modulating the EMT-signaling pathway in nude mice.

## Discussion

CRC is a common malignancy of the gastrointestinal tract, and although the main therapy options contain surgery, chemotherapy, and radiotherapy, the prognosis of CRC patients remains poor (27). With the evolution of sequencing technology in the past few decades, several biomarkers in CRC have been validated, which were of great significance for the diagnosis, prognosis, and treatment of patients with CRC. Hence, it is meaningful to identify core genes, which can be used as the promising biomarkers for the diagnosis, prognosis, and targeted therapy of CRC through multiomics and experimental analyses.

In the present study, a total of 218 upregulated and 326 downregulated DEGs were identified in the two gene datasets (*i*.*e*., GSE9348 and GSE110223). The results of functional enrichment analyses suggested that these DEGs might be involved in CRC pathogenesis and development. Prior research suggested that the inhibition of a high-fat diet activated fatty acid oxidation, creating a therapeutic opportunity to reverse the effects of a high-fat diet on intestinal tumorigenesis (28). In this study, the DEGs were enriched in the fatty acid metabolism pathway. Yang *et al*. (29) reported that *DOT1L* inhibition could induce cell cycle arrest at the S phase as well as downregulate the expression of *CDK2* and *CCNA2*. Similarly, the DEGs were enriched in the cell cycle pathway in the present study. *CDK1*, *CCNA2*, *CCNB1*, *CDC20*, and *TOP2A* were screened from the PPI network and considered core genes. Subsequently, they were identified to be highly expressed in CRC based on transcriptomics. *CCNA2* expression was associated with overall survival rates in CRC, suggesting a prognostic value of *CCNA2* in CRC. Furthermore, at the proteomics level, the CCNA2 protein level in the CRC tissues was higher than that in the normal colorectal tissues in this study. Previous studies have confirmed that *CCNA2* was elevated in various cancers (30). The latest research had shown that 5-year relapse-free survival was lower for CRC patients with *CCNA2*^nonhigh^ compared with CRC patients with *CCNA2*^high^ (14). *CCNA2* showed a diagnostic value in differentiating gastric cancer tissues from normal gastric tissues (31). In this study, *CCNA2* also showed an excellent diagnostic value in distinguishing CRC tissues from normal colorectal tissues. Furthermore, in the drug–gene interaction network, *CCNA2* interacted with traditional and novel anticancer drugs (tamoxifen and genistein). Tamoxifen could induce *CCNA2* upregulation in breast cancer cells, and genistein could induce G2 M phase arrest in breast cancer cell lines (32, 33). *CCNA2* is a potential diagnostic biomarker, prognostic indicator, and therapeutic target for CRC.

The correlation between *CCNA2* and immune infiltration was analyzed using immunomics. The *CCNA2* mRNA expression was correlated with the infiltration levels of B cells, CD8+ T cells, CD4+ T cells, neutrophils, dendritic cells, and macrophages. This might be related to the SCNA of *CCNA2* in CRC. Chen *et al*. (34) reported that all-*trans* retinoic acid inhibited signal transducer and activator of transcription 3 (*STAT3*) signaling, which downregulated *CCNA2* and PD-L1 expression, thereby playing an antitumor role in oral squamous cell carcinoma. In the present study, *CCNA2* was positively correlated with PD-L1 in CRC. We speculate that *CCNA2* is involved in the immune regulation of CRC. In the TF–mRNA network for this study, *TP53* was a transcriptional regulator upstream of *CCNA2*. *TP53*-WT endometrial cancer with high *CCNA2* expression had a targeted transcriptomic profile similar to that of *TP53*-mutant type endometrial cancer, suggesting that *CCNA2* was a seminal determinant for *TP53*-WT and *TP53*-mutant type endometrial cancer (35).

*CCNA2* is associated with cellular proliferation and can be used for molecular diagnostics as a proliferation marker (36). A previous study confirmed that cyclin A and/or the cyclin A–*CDK2* complex was a promising anticancer target with a high therapeutic index (37). At present, the role and mechanism of *CCNA2* in CRC have not been fully elucidated. A previous study revealed that compared with normal colonic epithelial cells, *CCNA2* was highly expressed at mRNA and protein expressions in different types of CRC cells, and *CCNA2* knockdown could lead to G2/M phase growth arrest in CRC cells (38). In a study on *CCNA2*-deficient transgenic mice, *CCNA2* expression in CRC mirrored distinct roles during colon carcinogenesis, such as driving cell proliferation in early stages when expressed highly but promoting aggressiveness in later stages when expressed slightly (12). *CCNA2* expression was lower in metastases relative to that in matched primary colon adenocarcinoma, suggesting that *CCNA2* negatively controlled motility by promoting RhoA activation, thereby showing a new function of *CCNA2* in cytoskeletal rearrangement and cell migration (39). In this study, *CCNA2* expression was upregulated in the CRC tissues and cells, and its downregulation could inhibit the proliferation, migration, and invasion of the CRC cells, as well as induce the apoptosis of CRC cells. Li *et al*. (40) reported that the involvement of *CCNA2* in the regulation of the EMT-signaling pathway was significantly associated with metastasis of bladder cancer. In the present study, the knockdown of *CCNA2* could suppress the EMT-signaling pathway in CRC. Hence, we hypothesize that *CCNA2* plays the role of a promoter in CRC pathogenesis and progression *via* regulation of the EMT-signaling pathway.

In the miRNA–mRNA network, miR-548x-3p showed a targeted regulatory relationship with *CCNA2*. Previous research found that miR-548x-3p was overexpressed in circulating monocytes of women with osteoporosis, which could inhibit the proliferation, migration, and invasion of SaOS2 and U2OS cells; the mechanism of miR-548x-3p as a modulator in bone remodeling might be related to the inhibition of MAF BZIP transcription factor B (*MAFB*) and signal transducer and activator of transcription 1 (*STAT1*) (41). In this study, the mRNA expression of miR-548x-3p was downregulated in the CRC tissues and cells. Kalhori *et al*. (42) reported that miR-548x overexpression could inhibit the proliferation of human glioblastoma cell lines, suggesting that miR-548x played an anticancer role in glioblastoma by controlling the PI3K–AKT signaling pathway. Similarly, miR-548x-3p overexpression could inhibit CRC cell proliferation in the present study. miR-548-5p could regulate tumor suppressor genes related to the cell cycle in endometrial carcinoma (43). In the present study, miR-548x-3p overexpression could induce the apoptosis of CRC. The downregulated miR-548x expression or upregulated *KIF2C* expression could rescue the invasive ability of bladder cancer cells, following circRGNEG silencing (44). In a study on esophageal squamous cell carcinoma, miR-548-3p and miR-576-5p could promote the migration and invasion of esophageal squamous carcinoma cells by inhibiting *NRIP1* expression (45). In this study, upregulation of miR-548x-3p could inhibit the migration and invasion of the CRC cells, whereas downregulation of miR-548x-3p could promote the migration and invasion of the CRC cells. We speculate that miR-548x-3p can act as a suppressor in CRC.

In prior research, *CCNA2* had been confirmed as one of the regulatory targets of miRNA. miR-31-5p regulated the proliferation, DNA synthesis, and apoptosis of human spermatogonial stem cells *via* the *PAK1*–*JAZF1*–*CCNA2* pathway (46). Waltonitone, an ursane-type pentacyclic triterpene extracted from *Gentiana waltonii* Burkill, recently appeared to exert an antitumor effect; waltonitone could suppress hepatocellular carcinoma cell proliferation and tumorigenesis *via* miR-22-regulated *CCNA2* repression and partially *via* farnesoid X receptor modulation (47). According to the correlation analysis in this study, miR-548x-3p was negatively correlated with *CCNA2* in CRC. The results of the dual luciferase reporter assay revealed that *CCNA2* was a direct target of miR-548x-3p in CRC. In CRC, the upregulated miR-548x-3p could downregulate *CCNA2* expression, whereas the downregulated miR-548x-3p could upregulate *CCNA2* expression. We infer that miR-548x-3p can regulate the *CCNA2* expression and shows a negative regulatory relationship in CRC. Furthermore, miR-548x-3p inhibits endogenous *CCNA2* expression in CRC.

A study on esophageal carcinoma showed that *CCNA2* downregulation could enhance the antitumor effects of miR-219-5p. *CCNA2* knockdown could significantly reduce the percentage of cells in the G0/G1 phase but increase the percentage of cells in the S and G2/M phases. This was similar to the effect induced by miR-219-5p overexpression (48). In ovarian cancer cells, *CCNA2* overexpression could rescue cell proliferation suppression mediated by miR-508-3p, suggesting that miR-508-3p could inhibit the proliferation of ovarian cancer cells *via* the targeted regulation of *CCNA2* (49). Restoring Tg737 significantly hampered miR-548a-5p-induced cell proliferation (50). The knockdown of *NEAT1*, a long noncoding RNA, inhibited cell growth and induced cell apoptosis in breast cancer cells; miR-548ar overexpression downregulated *NEAT1* expression and promoted apoptosis (51). The ability of miR-548x-3p to inhibit the proliferation, migration, and proliferation of the CRC cells was attenuated by *CCNA2* upregulation, whereas *CCNA2* overexpression could restore this ability. We conclude that *CCNA2* overexpression can lead to the dysregulation of the miR-548x-3p–*CCNA2* axis, thus affecting the biological characteristics of CRC cells. In a past study, *CCNA2* was targeted by miR-381-3p and consequently suppressed the EMT-signaling pathway (40). In this present study, restoring *CCNA2* could reverse the inhibitory effect of miR-548x-3p on the EMT-signaling pathway in CRC cells.

*CCNA2* is a regulator of the mitotic cycle and affects cell proliferation promoting G1/S and G2/M transitions of the cell cycle (52). In our study, the knockdown of *CCNA2* induced the apoptosis of tumor xenografts in nude mice. *CCNA2* was essential in both hematopoietic stem cells and embryonic stem cells but not in more differential mouse embryonic fibroblasts (53). CCNA2 protein was significantly decreased in tumor tissues harvested from miR-188-injected mice; miR-188 suppressed nasopharyngeal carcinoma initiation and progression *in vivo* potentially *via* the downregulation of genes involved in G1/S transition (54). In a study on liver cancer in transgenic mice with *CCNA2* conditional knockout, liver tumors in WT mice could be detected as early as 2 months, and all mice developed liver tumors within 4 months; for *CCNA2*^liv−/−^ (*CCNA2* knockout) mice, one of six mice developed liver tumors by 4 months, whereas the majority of them displayed liver tumors after 6 months (55). According to the tumorigenicity assay for the nude mice in this study, the knockdown of *CCNA2* could significantly inhibit the growth and proliferation of tumor xenografts, and it also could inhibit the EMT-signaling pathway in tumor xenografts. Hence, we hypothesized that *CCNA2* promotes the tumorigenesis of CRC cells *via* the EMT-signaling pathway and, subsequently, plays a carcinogenic role in CRC *in vivo*.

However, in this study, we were unsuccessful in elucidating the effect and mechanism of miR-548x-3p on CRC *in vivo*. The pharmacological mechanisms of *CCNA2* in the CRC need to be validated *in vitro* and *in vivo*. A transgenic animal model needs to be constructed to further validate the effect of the miR-548x-3p–*CCNA2* axis on CRC.

## Conclusion

To conclude, multiomics analysis revealed that *CCNA2* is a potential biomarker for the diagnosis, treatment, and prognosis of CRC and is associated with immune infiltration, TF, and miRNA. A novel regulatory role of the miR-548x-3p–*CCNA2* axis in regulating the tumorigenesis of CRC through the EMT-signaling pathway was identified in the present study *via* experimental analysis, thereby providing new sights and approaches to facilitate diagnosis, therapy, and prognosis of CRC.

## Experimental procedures

### Identification of core genes in CRC

Two gene datasets (*i*.*e*., GSE9348 and GSE110223) were obtained from GEO (https://www.ncbi.nlm.nih.gov/geo). GSE9348 included 70 CRC samples and 12 normal samples (56), whereas GSE110223 contained 13 CRC samples and 13 normal samples (57). GEO2R (https://www.ncbi.nlm.nih.gov/geo/geo2r) was used to identify DEGs in the two gene datasets, respectively. The DEGs with the same expression trend shared between the two gene datasets were screened out. The adjust *p* value (adjust *p*) <0.05 and |log_2_ fold change (log_2_ FC)| >1 were set as the threshold criterion. The Gene Ontology and Kyoto Encyclopedia of Genes and Genomes pathway analyses of DEGs were performed in DAVID (version 6.8, https://david.ncifcrf.gov/tools.jsp) (58, 59). The physical interactions and functional interactions of DEGs were analyzed in the search tool for the retrieval interaction genes (STRING, version 11.5, https://string-db.org) (60). The highest confidence (*i*.*e*., score of confidence >0.9) was considered to indicate statistical significance. The PPI network of DEGs was constructed by using Cytoscape (version 3.7.1, Cytoscape Consoritum). The top five genes ranked by the degrees in the PPI network were considered the core genes, which were then screened out. The mRNA expression and overall survival rates of core genes were analyzed in GEPIA (version 1.0, http://gepia.cancer-pku.cn), which is an online tool for analyzing data from TCGA and Genotype-Tissue Expression (GTEx) (61). Log-rank *p* < 0.05 and *p* (hazards ratio) <0.05 were set as the threshold criteria. The core genes with prognostic value in CRC were screened out. The protein expressions of a prognostic core gene on the normal tissues and CRC tissues were verified by using CAB000114 antibody in the human protein atlas (version 21.0; https://www.proteinatlas.org) (62). The expressions of the prognostic core gene were applied for receiver operating characteristic analysis to evaluate its diagnostic value to distinguish between the CRC tissues and normal tissues in the internal datasets (*i*.*e*., GSE9348 and GSE110223) and the independent external dataset (*i*.*e*., GSE74602). GSE74602 included 30 normal tissues and 30 CRC tissues. Receiver operating characteristic analysis was conducted in the RStudio by the pROC package (63). The prognostic core gene with the area under the curve >0.8 as well as *p* < 0.05 was set as the threshold criteria. The interaction between the prognostic core gene and the drugs was explored in the drug–gene interaction database (DGIdb, version 4.2.0-sha1 afd9f30b; https://dgidb.genome.wustl.edu), which could generate assumptions about the prognostic core gene that may be targeted therapeutically for drug development (64, 65). The drug–gene interaction network of the prognostic core gene was constructed by using Cytoscape (version 3.7.1, Cytoscape Consoritum).

### Bioinformatics analysis of the prognostic core gene in CRC

The correlation between infiltrating immunocytes and the prognostic core gene on CRC was analyzed in the Tumor Immune Estimation Resource (TIMER, version 1.0; https://cistrome.shinyapps.io/timer) by using the deconvolution method. The correlation between the SCNA of the prognostic core gene and infiltrating immunocytes was evaluated through the related modules; the relationship between the prognostic core gene and the gene markers of immune cells was explored *via* related modules (66). *p* < 0.05 was set as the threshold criteria. The miRnet (version 2.0; https://www.mirnet.ca) was utilized to identify TFs for prognostic core gene, which were based on the TRRUST database (tissue type: intestine) (67). The miRDB (https://mirdb.org) was applied to analyze the interaction between the miRNA and prognostic core gene, and the target score >60 was considered to indicate statistical significance (species: human) (68, 69). The TF–mRNA and miRNA–mRNA networks for the prognostic core gene were built by using Cytoscape (version 3.7.1, Cytoscape Consoritum). These results suggested that miR-548x-3p had a targeted regulatory relationship with *CCNA2*, which needs to be explored in the subsequent experiments.

### Samples and cell lines

A total of 13 paired human CRC tissues and their adjacent normal tissues were collected at the First Affiliated Hospital of Jinan University from CRC patients who did not receive any chemoradiotherapy before surgery. All participants provided written informed consent to take part in this study. This study was conducted with the approval of the Clinical Research Ethics Committees of the First Affiliated Hospital of Jinan University. The CRC cell lines (*i*.*e*., HCT116 [catalog no.: IM-H098], SW480 [catalog no.: IM-H111], and HT29 [catalog no.: IM-H102]) and normal human colonic epithelial cell line (*i*.*e*., NCM460 [catalog no.: IM-H445]) were purchased from Immocell Biotechnology Co, Ltd. All purchased cell lines were provided with short tandem repeat authentication and mycoplasma testing reports from the suppliers. In addition, we conducted a secondary mycoplasma contamination test using a mycoplasma detection kit 1 week after cell cultivation. All cell lines were maintained in Dulbecco's modified Eagle's medium (Gibco/Thermo Fisher Scientific) supplemented with 10% fetal bovine serum (Gibco/Thermo Fisher Scientific) at 37°C in a humidified atmosphere of 5% CO_2_.

### Immunohistochemistry

Paraffin slides of tissues were baked at 60°C for 1 h, deparaffinized with xylene, and rehydrated with an alcohol gradient. The tissue slides were immersed in 3% hydrogen peroxide for 10 min, and the antigens were retrieved in 10 mM sodium citrate buffer (pH 6.0), followed by heating at 100°C for 3 min. The tissue slides were blocked with 10% horse serum and then incubated at 37°C for 30 min. The slides were treated with primary antibody against *CCNA2* (1:500 dilution, #67955; Cell Signaling Technology), primary antibody against ki67 (1:500 dilution, #GB111499-100; ServiceBio) or primary antibody against caspase 3 (1:500 dilution, #GB115600-100; ServiceBio) at 4°C overnight. The tissue slides were then treated with the secondary antibody horseradish peroxidase goat anti-rabbit IgG (H + L) (1:200 dilution, AS014; Abclonal) at 37°C for 30 min. A DAB detection kit was used for visualization. Immunohistochemistry images were captured and analyzed by using ImageJ software (National Institutes of Health).

### RNA extraction and qPCR

TRIzol Reagent (#15596026; Thermo Fisher Scientific) was applied to extract the total cellular RNAs from the tissues and cells in accordance with the manufacturer’s instructions. The miRNA First Strand Complementary DNA (cDNA) Synthesis kit (TIANGEN Biotech) was used for miRNA reverse transcription.

The qPCR was conducted with the GeneAmp PCR System 9700 Thermocycler (Applied Biosystems) by using the TaqMan 2× Universal Master Mix (Thermo Fisher Scientific). The human *CCNA2* forward primer sequence was used as 5′-CACTGGTGGTCTGTGTTCTGTG-3′; the human *CCNA2* reverse primer sequence was 5′-ATGCCAGTCTTACTCATAGCTGA-3′. miR-548x-3p forward primer sequence was 5′-GCGTAAAAACTGCAATTAC-3′; and miR-548x-3p reverse primer sequence was 5′-CAGTGCGTGTCGTGGAGT-3′. The mRNA expressions of *CCNA2* and miR-548x-3p were normalized to GAPDH and U6, respectively. All qPCR reactions were repeated at least thrice.

### Western blotting

The proteins from the tissues and cells were isolated in radioimmunoprecipitation lysis buffer (#P0013; Beyotime Biotechnology). Then, the proteins were separated by using 10% SDS-PAGE (Millipore) and transferred onto the polyvinylidene difluoride (PVDF) membrane (Millipore). The PVDF membranes were blocked with 5% skim milk, and the PVDF membranes were incubated with primary antibodies *CCNA2* (1:1000 dilution, #67955; Cell Signaling Technology), E-cadherin (1:1000 dilution, #3195; Cell Signaling Technology), N-cadherin (1:1000 dilution, #13116; Cell Signaling Technology), GAPDH (1:3000 dilution, #AC002; Abclonal), and β-Actin (1:4000 dilution, #90422; SIGMA) at 4°C overnight. Next, the PVDF membranes were washed thrice with 0.1% TBS with Tween-20 and incubated with horseradish peroxidase–conjugated secondary antibody for 1 h at room temperature. The ECL kit (Millipore) was used for chemiluminescence protein detection.

### Construction and transfection

The gene sequence of human *CCNA2* (NM_001237.5) was downloaded for comparison. The cDNA was amplified through PCR to acquire the full-length *CCNA2*. The cDNA was evaluated by gene sequencing and then inserted into pLKO.1-U6-EF1a-copGFP-T2A-puro vector according to the manufacturer’s instructions (IGE). Cloning three CCNA2 gene knockdown sequences into the GV248 vector (IGE) and packaging the corresponding lentivirus.

The synthetic miRNA mimics and inhibitors of miR-548x-3p and scrambled NC RNAs (*i*.*e*., control mimic and control inhibitor) were bought from IGE. The cells were transfected using Lipofectamine 2000 (Invitrogen) according to the manufacturer’s instructions. The transfection medium was changed after 6 h of transfection to a medium supplemented with 10% fetal bovine serum, and the experiments were performed 48 h after transfection.

### CCK-8 assay

The CCK-8 assay was conducted to detect the proliferation ability of cells. Briefly, 100 μl of the cell fluid containing logarithmic-phase growing cells was cultured in a 96-well plate and incubated with the BPMI-1640 medium containing fetal bovine serum overnight at 37°C in a humidified atmosphere of 5% CO_2_. After the cells were transfected, the CCK-8 solution (CK04; Dojindo Molecular Technologies) was added at 24, 48, 72, and 96 h after transfection according to the manufacturer’s instructions. The absorbance was measured at 450 nm to evaluate the cell proliferation rates.

### Wound scratch assay

The wound scratch assay was conducted to detect the migration ability of cells. The transfected cells were inoculated into a 6-well plate and cultured until reaching 80% confluence. Then, scratches were drawn on the cell surface with sterile pipette tips, and the suspended cells were washed and cultured in a serum-free medium. The scratches were photographed at 0 and 24 h, respectively. Ultimately, the scratch wound healing rates were calculated.

### Transwell assay

The Transwell assay was conducted to detect the invasion ability of the cells. After the transfection of the cells, they were digested and counted by trypsin. The cells were suspended in a serum-free medium and added to the upper chamber of the kit, followed by culturing for 6 to 24 h (culture time was determined according to the cell state). After that, the cells were fixed with 4% paraformaldehyde for 20 min and stained with 0.1% crystal violet for 15 min. The nonmigrating/invading cells on the surface of the upper chamber were scraped with a cotton swab. The invaded cells were counted under an inverted microscope.

### Flow cytometry assay

The flow cytometry assay was performed to measure the cell apoptosis. The transfected cells were centrifuged and washed twice with precooled PBS, resuspended in 100 μl of 1× binding buffer, and then stained with Annexin V-FITC and propidium iodide kit (#A211-01; Vazyme) at room temperature for 15 min. The samples were then analyzed in an ACEA NovoCyte flow cytometer (ACEA Biosciences).

### Dual-luciferase reporter assay

The sequence of the WT and mutant type (MUT) *CCNA2* mRNA 3′-UTR with the binding site of miR-548x-3p were synthesized. When the cell density reached approximately 70%, WT and MUT were cotransfected with miR-548x-3p by using Lipofectamine 2000. The dual luciferase activity assays were examined by using the Dual Luciferase Reporter Assay System (Promega) according to the manufacturer’s instructions.

### Tumorigenicity in nude mice assay

A total of 10 male BALB/c-nu/nu mice (aged 4–6 weeks, weight: 15–20 g) were purchased from the laboratory animal center of the Sun Yat-Sen University and maintained in the Zhejiang Cancer Hospital–accredited animal facility. All animal studies were performed in accordance with the National Institutes of Health animal-use guidelines and a protocol approved by the Ethics Committee of the Animal Center of the Zhejiang Cancer Hospital. All nude mice, in accordance with the random number table, were randomly assigned to the following groups: sh-NC (control group, n = 5) and sh-*CCNA2* (*CCNA2*-knockdown group, n = 5).

Each group of nude mice was injected with HCT116 cells, and a single cell suspension (1 × 10^7^ cells/ml) was injected subcutaneously into the back of the nude mice with a disposable aseptic syringe. The largest diameter (A) and its perpendicular (B) were measured once with a Vernier caliper on every fifth day. The tumor volume was calculated using the following formula: A × B^2^/2. The nude mice were euthanized 28 days later, and the tumor xenografts were extracted from the nude mice. Then, the isolated tumor xenografts were weighed. The tumor xenografts from nude mice were used to extract the protein, followed by Western blotting to detect the protein expressions of CCNA2, E-cadherin, and N-cadherin. The tumor xenografts were stained with H&E. The expressions of CCNA2, ki67, and caspase 3 in tumor xenografts were detected *via* immunohistochemistry.

## Statistical analyses

Statistical analysis was performed by using the GraphPad Prism 5.0 (GraphPad Software Inc) and SPSS 22.0 software (International Business Machines Corporation). Data were represented as the mean ± SD. Student *t* tests were performed for comparison between the two groups. One-way ANOVA was utilized for multiple group comparisons. Ranked data were analyzed by Wilcoxon’s rank-sum test. Spearman rho and Pearson correlation coefficient were used to evaluate the correlation of statistical data. *p* < 0.05 indicated that the difference was statistically significant.

## Ethics approval and consent to participate

The assay on human subjects was under the consent of the Clinical Research Ethics Committees of the First Affiliated Hospital of Jinan University. The assay on human subjects was carried out in accordance with the Declaration of Helsinki. All protocols of animal studies were approved by the Ethic Committee of the Animal Center of Zhejiang Cancer Hospital. All applicable international, national, and institutional guidelines for the care and use of animals were followed.

## Consent for publication

All CRC patient materials were provided written informed consent to take part in this study.

## Data availability

The datasets analyzed in this study can be found in GEO (https://www.ncbi.nlm.nih.gov/geo) and TCGA (https://portal.gdc.cancer.gov). The datasets used and analyzed in this study are available from the corresponding author upon reasonable request.

## Supporting information

This article contains supporting information (62).

## Conflict of interest

The authors declare that they have no conflicts of interest with the contents of this article.

## Figures and Tables

**Figure S4:**