# Supplementary material for: Exploring the regulatory mechanism of CCNA2 in colorectal cancer: Insights from multiomics and experimental analysis
Source: J Biol Chem. 2025 May 8;301(8):110216. doi: 10.1016/j.jbc.2025.110216 (PMC12319254; doi:10.1016/j.jbc.2025.110216)
Supplement: Supporting Information [file mmc1.docx]

**Supplement Figure 1**

Identification of differentially expressed genes (DEGs) between CRC tissues and normal tissues in gene datasets. (A). 218 upregulated genes were expressed in the GSE9348 and GSE110223 with log_2_ FC > 1 and adjusted P < 0.05. (B). 326 downregulated genes were expressed in the GSE9348 and GSE110223 with log_2_ FC < -1 and adjusted P < 0.05.

**Supplement Figure 2**

The Gene Ontology (GO) and Kyoto Encyclopedia of Genes and Genomes (KEGG) pathways of DEGs in database for annotation, visualization, and integrated discovery (DAVID). The GO terms and KEGG pathways were ranked according to -log_10_ (P Value), and the top 5 terms were selected. The GO terms included biological process term, cellular component term, and molecular function term. Gene Counts: the number of enriched DEGs in each term. Gene Ratio: the ratio of the number of enriched DEGs in each term to the total number of DEGs.

**Supplement Figure 3**

The protein expression of the prognostic core gene was validated in HPA (Version 21.0, [https://www.proteinatlas.org](https://www.proteinatlas.org/)). The protein expressions of the prognostic core gene were assigned four groups: high, medium, low, and undetected based on the scoring system, which contained the proportion of stained cells (i.e., > 75%, 25-75%, and < 25%) and the intensity of staining (i.e., strong, moderate, weak, and negative).

**Supplement Figure 4**

Construction of stable cell lines with knockdown of *CCNA2* expression. (A). The fluorescence imaging of HT29 stable cell line with knockdown *CCNA2* expression. (B). The fluorescence imaging of HCT116 stable cell lines with knockdown *CCNA2* expression.

**Supplement Table 1**

Identification of DEGs in colorectal cancer tissues compared with normal tissues

**Supplement Table 2**

Topology properties of core genes
