# Supplementary material for: Exploring the regulatory mechanism of CCNA2 in colorectal cancer: Insights from multiomics and experimental analysis
Source: J Biol Chem. 2025 May 8;301(8):110216. doi: 10.1016/j.jbc.2025.110216 (PMC12319254; doi:10.1016/j.jbc.2025.110216)
Supplement: Table S1 [file mmc2.docx]

**Supplement Table 1 Identification of DEGs in colorectal cancer tissues compared with normal tissues**

| **DEGs** | **Gene Names** |
| --- | --- |
| up regulated | *CDH3, TEAD4, INHBA, GTF2IRD1, CSE1L, DUSP14, CXCL8, SLC7A5, MIR664B//SNORA56//DKC1, RPN2, CEMIP, S100A11, NFE2L3, XPOT, NEBL, UBE2C, CBFB, COL11A1, MYC, PUS7, UTP14A, DPEP1, PPM1H, TPX2, TRIP13, RFC3, ECT2,* ***TOP2A****, SOX9, MRPS17, TOMM34, CKAP2, SLCO4A1, SORD, NPM1, SQLE, RNF43, UBE2S, CDK4, IFITM3, EIF2S2, SOX4, CXCL3, OSBPL3, GDF15, AHCY, ADGRG1, TGFBI,* ***CDK1****, PHLDA1, PPAT, AURKA, CCT2, PSAT1, NELFCD, CEP55, PAICS, HSP90AB1, ATP11A, CCT6A, MET, CTPS1, MSH6, KIF4A, HSPH1, DDX21, FASN, RPP40, NUFIP1, TTK, GGCT, HILPDA, MTHFD2, PRMT1, TKT, HMGB1, SLC35F6//CENPA, CXCL2, GTF3A, BGN, CKS2, GPSM2, SLC7A1, CXCL1, CDC25B, CCND1, IFITM2//IFITM1, THBS2, IARS, FAP, ASPM, IFITM1, KRT23, NME1, IPO5, FOXM1, NCAPG2, LY6G6F//LY6G6D, S100P, TIMP1, PFDN4, KPNA2, FANCI, VARS, BHLHE40, TESC, IFITM2, ATAD2, FABP6, PNO1, RRM2, MMP7, PRDX4,* ***CCNB1****, COL1A2, TMEM97, SHMT2, GTPBP4, MIF, CENPF, SLC25A32, TFDP1, CENPN, ACLY, TDGF1P3//TDGF1, IPO7, COL4A1, MAD2L1, SCD, FEN1, STC1, SRPX2, AZGP1, CST1, HS2ST1, NEK2, MCM2, HSPD1, PMAIP1, GINS1, COL1A1, MIR7112//BOP1, TRIM29, SLC12A2, NUSAP1, EEF1E1, SYNCRIP, CDKN3, TM9SF4, PLS3, UCHL3, CD44, PTTG1, MCM7, MELK, TCFL5, MKI67, VEGFA, MMP3, TM4SF1,* ***CCNA2****, DHCR7, MCM3, SLC7A11, S100A2, CDC6, EDNRA, ABCE1, DLGAP5, NT5DC2, NONO, EREG, LAPTM4B, LSM5,* ***CDC20****, SULF1, DDIT4, RANBP1, MORC4, HACD3, MMP11, DTL, PRKDC, PLA2G16, MCM4, BUB1B, DACH1, COL10A1, VSNL1, LOXL2, PRC1, COL5A2, SFRP4, PLAU, BACE2, RTEL1-TNFRSF6B//TNFRSF6B, POLR1D, MRGBP, TACSTD2, EPB41L2, SERPINE2, VCAN, MSH5-SAPCD1//SAPCD1//MSH5, MMP1, CFB, MRPS12, LGR5, CDH11, KLK10, SETSIP//SETP4//SET, PLCB4, KRT6B, SPP1, REG1A, MMP12, CANX, REG1B , SERPINB5.* |
| down regulated | *GUCA2B, HIGD1A, PHLPP2, GRAMD3, ABCG2, UGP2, APPL2, TRPM6, SLC4A4, PLCE1, GBA3, GUCA2A, RIOK3, PRDX6, SLC30A10, PDE8A, HPGD, RETSAT, CCDC68, CASP7, ETFDH, IL6R, AHCYL2, CD177, SLC22A5, ETHE1, CA1, EMP1, SLC17A4, AQP8, SLC36A1, SLC25A20, MIR22//MIR22HG, UGDH, MIR4680//PDCD4, CA12, FUCA1, CLMN, ZZEF1, LRRC19, MEP1B, PAPSS2, ACADS, C4orf19, CA7, NR1H4, CEACAM1, CNNM4, NAAA, P2RX4, C1orf115, ENTPD5, DHRS9, ENDOD1, EPB41L3, PLPP1, DHRS11, CA4, TDP2, SYNE3//LINC00341, GALNT12, AKR1B10, MALL, TUBAL3, HSD17B2, MS4A12, TSPAN7, HHLA2, SMPDL3A, ADTRP, ANK3, SPIB, STYK1, SMPD1, SLCO2A1, SULT1A2, GPD1L, GNA11, CLCA4, KLF4, CA2, ARL14, ABCA8, ACOX1, CCL15-CCL14//CCL15, TRANK1, HSD17B11, IL10RB, NAT1, ACAA2, SRI, HSD11B2, TP53I3, MXI1, PBLD, CITED2, DYRK2, CLIC5, TSPAN1, TLR3, PYY, ANPEP, SQRDL, PDZD3, EDN3, SLC1A1, TST, ABHD3, TNFSF10, 8-Mar, CLDN8, MT1F, SULT1A1, CDHR5, RNF125, PTP4A1, CEACAM7, SPINK5, CWH43, MYO1A, SLC35D1, PLAC8, NR3C2, UGT1A3//UGT1A1//UGT1A4//UGT1A9//UGT1A5//UGT1A6//UGT1A7//UGT1A8//UGT1A10, PTPRH, GDPD3, SCNN1B, EGLN3, ADAMDEC1, NR5A2, MT1E, MT1M, IGH, EPB41L4B, GPA33, MGLL, SULT1B1, FGL2, SLC26A2, TMPRSS2, UGT1A3//UGT1A1//UGT1A9//UGT1A5//UGT1A8, AK1, CFD, DPT, C7, CLU, FNBP1, CHP2, NTRK2, CCDC69, HBB, TARP///TRGV9///TRGC2, PRKACB, LOC100652777///PLA2G10, LPAR1, UGT1A1//UGT1A4//UGT1A9//UGT1A6//UGT1A8//UGT1A10, KAT2B, RAB27A, RCAN1, FMO5, PLCL2, SECTM1, ZG16, FXYD3, CDHR2, PGM1, ADCY9, FAM47E-STBD1///FAM47E///STBD1, CPT1A, ABCA5, IL1R2, LOC339166///WSCD1, OSBPL1A, STAP2, PTPRR, AOC1, ASPA, STMN2, ADH1B, MAOA, MT1X, MT1G, FGFR2, HMGCS2, DNASE1L3, METTL7A, BMP2, GCG, LRMP, CPM, ACAT1, SGK2, CES3, NEDD4L, LINC00483, FTH1, BCHE, MT2A, BTNL8, PLSCR4, IQGAP2, GCNT3, PADI2, RCAN2, NR3C1, PIP5K1B, SI, EPHX2, CXCL12, LGALS2, TNXB//TNXA, PIGZ, LRRN2, ENPP2, PRKCB, MT1HL1, ITM2A, ITM2C, BCL2, MEF2C, ATP8A1, MT1H, KRT20, ADAM28, CLCA1, NXPE4, AKAP7, ANK2, GPX3, ADH1C, PCK1, SEMA6A, NAALADL1, SLC26A3, BEST2, ECI2, TNFRSF17, TJP3, MEP1A, PDE9A, FAM107A, SGK1, OPN3, SELENBP1, GREM2, GHR, BDKRB2, PLXNA2, CHGA, VILL, GZMA, A1CF, BTBD3, SRPX, KCNMA1, LOC100506558//MATN2, SLC20A1, EXOSC7//CLEC3B, FCGBP, MS4A1, PARM1, FHL1, ATP2A3, CD48, P2RY14, SEPP1, FAM30A, GSN, APOBR, PID1, PTGS1, PTPRC, CCL19, VIPR1, CDA, ACACB, DEFB1, PPP1R14D, MUC4, MYO15B, FAM129A, ABCB4//ABCB1, IGLJ3//IGLV@//IGLC1, CD52, FRZB, IGHM, POU2AF1, HIST1H1C, PTGER4, SLC3A1, JCHAIN, CKB, IGLC1, SST, RARRES3, CACNB2, LOC100507472//PCSK6, ABCB1, SPARCL1, CCL5, CR2, IGLV1-44, FAM134B, IGK//IGKC, MYH11, TRBC1, IGKC, FCMR, TBC1D9, FABP4, MUC2, KIT, GPM6B, CHRDL1, FABP1, IGHA2//IGHA1//IGH, SFRP1, CXCL13, MYLK, BHLHE41, APOBEC3B, UGT2B15, UGT2B17, CYAT1//IGLV1-44//IGLC1, ALDH1A1, CD37.* |

218 up regulated DEGs and 326 down regulated DEGs were identified in colorectal cancer tissues, compared with normal tissues. The core genes were shown in boldface.
