# Supplementary material for: Exploring the regulatory mechanism of CCNA2 in colorectal cancer: Insights from multiomics and experimental analysis
Source: J Biol Chem. 2025 May 8;301(8):110216. doi: 10.1016/j.jbc.2025.110216 (PMC12319254; doi:10.1016/j.jbc.2025.110216)
Supplement: Table S2 [file mmc3.docx]

**Supplement Table 2 Topology properties of core genes**

| Genes | Degree | Betweenness centrality | Closeness centrality | Clustering coefficient | Stress | Average shortest path length |
| --- | --- | --- | --- | --- | --- | --- |
| *CDK1* | 45 | 0.23 | 0.41 | 0.39 | 12244 | 2.45 |
| *CCNA2*  *CCNB1*  *CDC20*  *TOP2A* | 39  38  35  32 | 0.05  0.16  0.02  0.01 | 0.36  0.40  0.34  0.35 | 0.48  0.47  0.57  0.65 | 4758  9260  2344  1864 | 2.78  2.51  2.90  2.88 |

The core genes were ranked by degree.
